# Characterization of Microbial Communities in Pilot-Scale Constructed Wetlands with *Salicornia* for Treatment of Marine Aquaculture Effluents

**DOI:** 10.1155/2018/7819840

**Published:** 2018-04-29

**Authors:** Xiaona Ma, Xingqiang Song, Xian Li, Songzhe Fu, Meng Li, Ying Liu

**Affiliations:** ^1^Key Laboratory of Experimental Marine Biology, Institute of Oceanology, Chinese Academy of Sciences, Qingdao 266071, China; ^2^Laboratory for Marine Fisheries Science and Food Production Processes, Qingdao National Laboratory for Marine Science and Technology, Qingdao 266235, China; ^3^University of Chinese Academy of Sciences, Beijing 100039, China; ^4^Faculty of Biosciences, Fisheries and Economics, The Arctic University of Norway, 9037 Tromsø, Norway; ^5^School of Marine Science and Environmental Engineering, Dalian Ocean University, Dalian 116023, China; ^6^Fisheries College, Ocean University of China, Qingdao 266001, China

## Abstract

Microorganisms play an essential role in the performance of constructed wetlands (CWs) for wastewater treatment. However, there has been limited discussion on the characteristics of microbial communities in CWs for treatment of effluents from marine recirculating aquaculture systems (RAS). This study is aimed at characterizing the microbial communities of pilot-scale CWs with *Salicornia bigelovii* for treatment of saline wastewater from a land-based Atlantic salmon RAS plant located in Northern China. Illumina high-throughput sequencing was employed to identify the profile of microbial communities of three CWs receiving wastewater under different total ammonia nitrogen (TAN) concentrations. Results of this study showed remarkable spatial variations in diversity and composition of microbial communities between roots and substrates in three CWs, with distinct response to different TAN concentrations. In particular, Proteobacteria, Firmicutes, Cyanobacteria, and Bacteroidetes were predominant in roots, while Cyanobacteria, Proteobacteria, Firmicutes, Verrucomicrobia, and Bacteroidetes were prevalent in substrates. Moreover, redundancy analysis indicated that specific functional genera, such as *Nitrosopumilus*, *Vibrio*, *Pseudoalteromonas*, *Nitrospina*, and *Planctomyces*, played key roles in the removal of nitrogen/phosphorus pollutants and growth of wetland plants. From a microorganism perspective, the findings of this study could contribute to better understanding of contaminants' removal mechanism and improved management of CWs for treatment of effluents from land-based marine aquaculture.

## 1. Introduction

Development of environment-friendly and efficient aquaculture effluent treatment system is crucial for sustainable intensification of aquaculture, including recirculating aquaculture systems (RAS). Due to large volumes of wastewater with high salinity, it remains a challenge for treatment of effluents from land-based marine aquaculture. A number of physical (e.g., mechanical filtration [1]), chemical (e.g., catalytic reduction [2]), and biological (e.g., periphyton biofilters [3]) methods, used in conventional wastewater treatment, have been applied for treating mariculture wastewater, while they are costly in terms of capital investment, energy demand, and system maintenance [4]. Alternatively, constructed wetlands (CWs) act as a natural biofilter and can remove considerable amounts of nutrients, organic matter, and suspended solids from wastewater [5, 6]. Owing to low capital, operating costs, and low energy consumption, CWs are becoming a promising technique to treat aquaculture effluents before discharge.

The performance of CWs largely depends on the interaction of wetland substrates, plants, and their associated microorganisms [7]. In particular, microorganisms within the biofilm on the surface of filter media and plant roots are widely considered to play a key role in the removal of many organic and inorganic pollutants [8, 9]. In recent years, a growing body of literature has examined the response of microbial community in CWs to wastewater quality characteristics [9], substrate type [10], plant diversity [11], pH variation [12], operational time [13] and so on. In a generic context, a better understanding of microbial communities in CWs and their influential parameters could aid in optimization and management of CWs toward further efficiency enhancement [14, 15]. Until now, only a few published studies have focused on CWs for treatment of saline wastewater from offshore and coastal marine aquaculture [16], while the characteristics of microbial communities in CWs for mariculture wastewater treatment have not yet been dealt with in depth.

A number of methods are available for the assay of environmental microbial characteristics, for example, plate count method, machine learning-based measurements, and molecular technologies [7, 17]. High-throughput sequencing technology is a highly efficient molecular biology method to profile complicated microbial populations of CWs [10, 18, 19], which provides an opportunity to investigate the links between the microbial communities and operational environment of CWs in particular [15, 20]. Recently, Urakawa and Bernhard [21] emphasized further research on high-throughput sequencing of wetland microbial communities to support the potential use of microorganisms as effective biological indicators for wetland management. To date, there are few published studies on the characteristics of microbial communities in CWs treating mariculture effluents, based on the high-throughput sequencing technology.

The aim of this study was to characterize the diversity and structure of microbial communities attached to substrate surface and plant roots in CWs with *Salicornia* spp. for treatment of mariculture wastewater under three different total ammonia nitrogen (TAN) concentrations, using Illumina high-throughput sequencing method. Moreover, the contributing microorganisms and core genera to the removal of nitrogen and phosphorus from wastewater were identified, and the relationships between nutrients' removal efficiency and corresponding functional genera were investigated.

## 2. Materials and Methods

### 2.1. Experimental Wetland System

**Figure 1:**
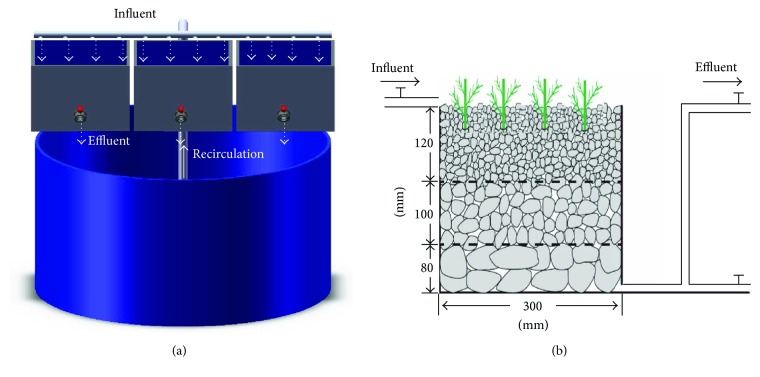
The pilot CW system (a) and a single CW unit (b). (Figure (b) was adapted from Li et al. (unpublished data) [70]).

Three pilot-scale recirculating horizontal subsurface flow CW systems (Figure 1) were constructed to treat simulated wastewater from a land-based intensive Atlantic salmon (*Salmo salar*) farm, located in Shandong Province, Northern China. Each CW system had one cylindrical barrel (diameter (Ø), 900 mm; height, 670 mm) and three respective CW tanks (300 mm × 300 mm × 300 mm, W × L × H). Each CW tank was filled with graded smooth cobblestone (Ø, 30–50 mm; height, 80 mm) as the bottom layer, haydite (Ø, 5–8 mm; height, 100 mm) as the middle layer, and smaller haydite (Ø, 3–5 mm; height, 120 mm) as the top layer. A total of 12 *Salicornia* plants (fresh weight, 2.0 ± 0.1 g/plant) were planted in each tank. Before the experiment, the *Salicornia* plants were, first, subjected to salt acclimation for 30 days for adaptation to the salinity of seawater used in this farm and then moved to the CWs and fed with seawater in batches for 60 days.

Fermented with Atlantic salmon residual excrement bait [22], the simulated wastewater was diluted to different TAN concentrations and classified into three groups, namely, low-concentration group (L, 0.75 ± 0.01 mg/L), middle-concentration group (M, 2.31 ± 0.09 mg/L), and high-concentration group (H, 7.23 ± 0.18 mg/L), representing the range of observed TAN concentrations in actual wastewater from the salmon farm under study. The simulated wastewater was stored in the barrel and then pumped by peristaltic pumps to the CW system (each with three parallel CW units). The outflows of the CWs went back to the barrel by gravity. Wastewater in the barrel was completely replaced every 18 days. During the experimental period, wastewater flowed into the CWs at a rate of 100 mL/min. Before sample collection, all the three CWs were in operation continuously for 72 days. At the end of the experiment, sample collection and monitoring of influent and effluent wastewater quality were performed.  Table 1 presented the effluent wastewater characteristics and removal performance of the CWs, including TAN, nitrite (NO_2_^−^-N), nitrate (NO_3_^−^-N), phosphate (PO_4_^3−^-P), temperature (T), and pH. The removal performance was expressed by final variation and removal rate. By the end of the experiment, the fresh weight of the harvested *Salicornia* plants (g/plant) in the three CWs was 10.0 ± 1.4 (low-concentration group), 12.8 ± 3.6 (middle-concentration group), and 9.8 ± 3.9 (high-concentration group).

### 2.2. Sample Collection and DNA Extraction

In order to achieve the maximum recovery rate and representative information on microbial populations, samples were collected both from the plant roots (R-samples) and the substrate (S-samples) surface on several selected spots of each experimental wetland system. In total, nine R-samples (1 g·ind^−1^) were collected, including three from the L group (L-R, replicate samples marked as L1-R, L2-R, and L3-R), three from the M group (M-R, replicate samples marked as M1-R, M2-R, and M3-R), and three from the H group (H-R, replicate samples marked as H1-R, H2-R, and H3-R). Similarly, nine S-samples (10 g·ind^−1^) were collected from the top layer of the CWs, including three from the L group (L-S, replicate samples marked as L1-S, L2-S, and L3-S), three from the M group (M-S, replicate samples marked as M1-S, M2-S, and M3-S), and three from the H group (H-S, replicate samples marked as H1-S, H2-S, and H3-S).

The attached biofilms on the R- and S-samples were extracted by means of shaking each sample in 100 mL of sterile physiological saline with 100 *μ*L of Tween 80 detergent solution using a vortex mixer for 10 min. Then, the solution was filtered through a 0.22 *μ*m polycarbonate filter (Millipore, MA, USA) to collect the microorganisms. All the processed samples were stored at −80°C until microbial DNA extraction. The total DNA on the filter paper was extracted with E.Z.N.A.® Water DNA Kit (Omega Bio-Tek, Norcross, GA, USA) according to the manufacturer's protocol. Thereafter, the extracted DNA was subjected to electrophoresis using 1.0% agarose gel at 150 V for 20 min to examine the quality of DNA. DNA purity and quantity were determined using a NanoDrop spectrophotometer (NanoDrop Technologies Inc., Wilmington, DE, USA). The extracted DNAs were stored at −80°C before being subjected to high-throughput sequencing.

### 2.3. High-Throughput Sequencing

Deep sequencing of the 16S rRNA gene amplicons from the 18 samples was performed using Illumina MiSeq paired-end sequencing platform (Illumina, San Diego, CA, USA). First, polymerase chain reaction (PCR) was carried out using 25 *μ*L of reaction mixture containing 1x PCR buffer, 10 ng of genomic DNA, 0.5 U of Ex Taq (Takara, Dalian, China), 1.5 mmol/L MgCl_2_, 0.4 *μ*mol/L deoxynucleoside triphosphate (dNTP), and 1.0 *μ*mol/L each primer. The primer pair used for PCR was 515F (5′-GTGYCAGCMGCCGCGGTA-3′) and 909R (5′-CCCCGYCAATTCMTTTRAGT-3′), targeting the V4-V5 hypervariable region of bacterial 16S rRNA gene [23, 24]. The PCR profile consisted of initial denaturation at 94°C for 3 min, followed by 30 cycles of denaturation at 94°C for 40 s, annealing at 56°C for 60 s, elongation at 72°C for 60 s, and final extension at 72°C for 10 min. Each sample was amplified in duplicate and then combined together. The PCR products were separated by electrophoresis on 1.2% agarose gel and purified using SanPrep DNA Gel Extraction Kit (Sangon Biotech, Shanghai, China). The DNAs in the PCR products were quantified with NanoDrop, and amplicons from each sample were pooled at equimolar ratios based on the DNA concentration. The purified mixtures were finally sequenced on the Illumina MiSeq platform.

### 2.4. Statistical Analyses

The raw data obtained from the Illumina MiSeq paired-end sequencing platform were merged with FLASH [25]. After quality filtering, the merged sequences were screened and filtered for quality and length using QIIME 1.9.0 [26]. Clean sequences (length> 300 bp, without ambiguous base “N” and average base quality score > 30) were checked and filtered using UCHIME program to remove chimeric sequences [27], and effect sequences without chimera were clustered into operational taxonomic units (OTUs) at 97% similarity. Representative sequences processed with QIIME 1.9.0 were used for taxonomic assignments based on Ribosomal Database Project classifier [28] and Greengenes database [29]. In order to compare the microbial communities of the collected samples, alpha diversity indices were obtained using QIIME 1.9.0 package, including Chao 1 richness estimator, Shannon index, and Simpson index. While visualizing the differences in the microbial community structure among the samples, a hierarchical cluster heatmap was generated and principal coordinate analysis (PCoA) on weighted and unweighted UniFrac distances of the 16S rRNA genes was performed with the R package vegan. Moreover, redundancy analysis (RDA) was conducted, using Canoco version 5.0, to explore all possible correlations between functional genera and nutrients' removal effect in the CWs. All other statistical analyses were made using SPSS version 13.0 along with Student's *t*-test and one-way analysis of variance (ANOVA), with significant difference set at *p* < 0.05.

## 3. Results and Discussion

### 3.1. Analysis of Sequence Data and Alpha Diversity

In this study, a total of 306,489 high-quality 16S rRNA gene sequence reads were obtained from the 18 samples subjected to Illumina MiSeq sequencing. Each library contained 9989–39,691 reads that were normalized to 9989 for comparison of microbial community diversity. The alpha diversity indices (OTU number, Chao 1 index, Shannon index, and Simpson index) were calculated for comparison of the microbial community richness and diversity between the R- and S-samples collected from the three CWs (Table 2).

Results of this alpha diversity analysis showed that the microbial population on the plant roots had higher community richness and diversity compared to the substrate surface. As seen in Table 2, all of the four alpha diversity indices for the R-samples, especially the Chao 1 index of the R-samples from the M group (*p* < 0.05), were higher than those of the S-samples from the three CWs. The OTU number and Chao 1 index [30] were used to analyze the microbial community richness of the R- and S-samples from the three CWs. In total, 32,670 OTUs were generated with a threshold of 0.97. Based on the OTU results (ranging from 1492 ± 274 to 2089 ± 202 (mean ± standard deviation)), the samples were ranked as L-S < M-S < H-S < M-R < L-R < H-R. Based on the Chao 1 index (average, varying from 6557 ± 685 to 9759 ± 1750), the samples were ranked as M-S < L-S < H-S < H-R < L-R < M-R. Furthermore, Shannon and Simpson indices were employed to analyze diversity and evenness of microbial species [31, 32]. The results of Shannon index were as follows: L-S (5.51 ± 1.20) < M-S (5.70 ± 0.90) < H-S (6.50 ± 1.03) < M-R (7.34 ± 0.85) < L-R (7.41 ± 0.78) < H-R (7.85 ± 0.71), which were similar to those of Simpson index (ranging between 0.86 ± 0.08 and 0.97 ± 0.02). Moreover, the relatively small standard deviation of the alpha diversity results within each treatment group indicated a good reproducibility of our experiments.

Results of this study indicated a remarkable spatial variation in the microbial community richness and diversity in the CWs. These results reinforce previous findings in the literature on spatial diversity of microbial communities. For example, Urakawa et al. [33] demonstrated that rhizosphere attracts microbial cells and maintains larger microbial diversity indices than the biofilm on substrate in a floating treatment wetland. Differently, results of the alpha diversity indices obtained in the present study are slightly higher than those reported in previous studies [10, 20], probably owing to varying operational factors of CWs (e.g., plant species, hydraulic loading rates, and wastewater characteristics) in those studies.

### 3.2. Comparison of Microbial Community Structures

**Figure 2:**
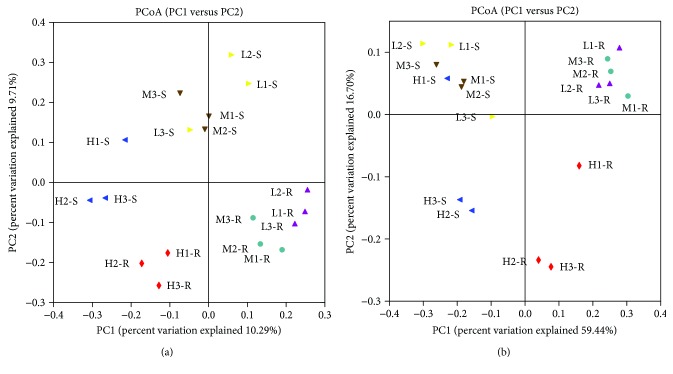
Unweighted (a) and weighted (b) principal coordinate analysis (PCoA) of the R- and S-samples from the three CW groups treating mariculture wastewater with different TAN concentrations.

Results of hierarchical cluster heatmap analysis of the microbial communities at genus level (Figure S1, Supplementary Material) and PCoA based on weighted and unweighted UniFrac distances (Figure 2) served as a basis for analysis of the relationships of microbial communities among the three different CWs. As seen from the heatmap and PCoA, good reproducibility of our experiments could be speculated from the result that three parallel samples in every treatment group were clustered together. In specific, all the R-samples were clustered in the left subgroup and all S-samples were clustered in the right subgroup, which indicated different microbial communities and a remarkable spatial variation between the plant roots and substrate surface. Furthermore, all the R-samples were gathered into three distinctive clusters according to different TAN concentrations, showing that they harbored different microbial communities. For the S-samples, most of them were tightly clustered by TAN concentrations, though they were not well grouped. This result demonstrated that microbial communities both on plant roots and substrate were influenced by the TAN concentrations of the CW inflows. The principal component axes PC1 and PC2 accounted for 59.44% and 16.70% of the total changes in the bacterial community structure, respectively.

This study revealed a spatial variation in the microbial communities on the roots and substrate, which might be attributed to oxygen diffusions and secretions from root. It is interesting to note that the oxygen concentration differs between the root and substrate areas because of root respiration and plant mechanisms for transporting oxygen to the rhizosphere [33]. For instance, Ansola et al. [20] reported that the microbial community gradient from flooded areas (lagoon) to dry-wet areas (zones with plant) was different and possibly related to oxygen concentration (from oxygen-poor flooded areas to dry areas with higher oxygen diffusivity). Haichar et al. [34] suggested that nutrient compounds and/or allelochemicals as root exudate could control microbial populations.

Results of this study, as mentioned above, showed that TAN concentrations of the CW inflow affected the microbial communities both on plant roots and substrate. This result was consistent with previous findings on the impact of TAN concentrations on microbial community, especially on ammonia-oxidizing prokaryote community [35, 36]. For instance, Shen et al. [35] found that nitrogen inputs significantly altered ammonia-oxidizing prokaryote community, with the influence varying among different systems. According to Urakawa et al. [36], ammonia availability is a major factor that determines the distribution of ammonia-oxidizing prokaryotes in coastal water.

### 3.3. Composition of Dominant Microbial Population

**Figure 3:**
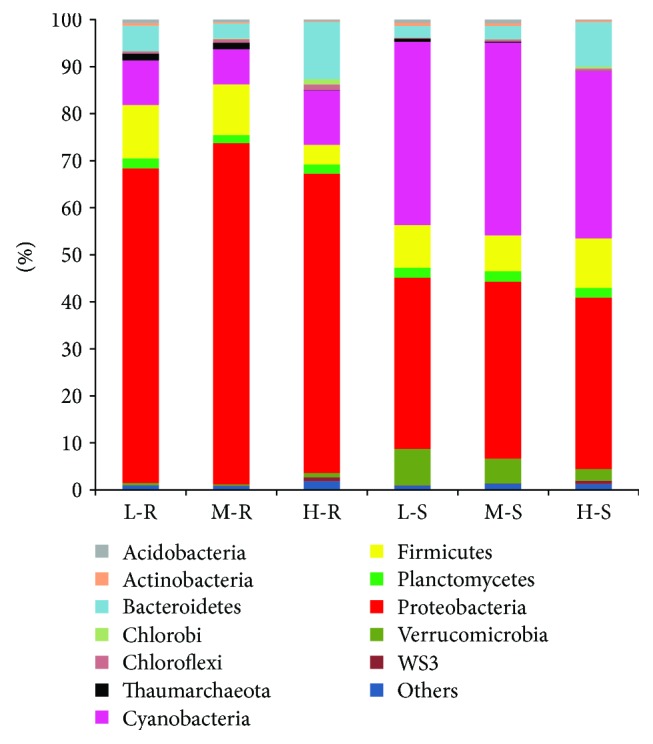
Relative abundance of microorganisms at the phylum level. “Others” refers to the sum of rare taxa each < 0.5% of the total.

Microbial phylum with a detection frequency of >0.5% in one or more samples was defined as a dominant phylum in this study. A total of 12 phyla (11 bacterial phyla and 1 archaeal phylum) were identified among the 18 samples (Figure 3), including Proteobacteria, Firmicutes, Cyanobacteria, Bacteroidetes, Planctomycetes, Thaumarchaeota (archaea), Acidobacteria, Actinobacteria, Verrucomicrobia, Chloroflexi, WS3, and Chlorobi. Only a small proportion of sequences (0.88–1.87%) retrieved from the three CWs could not be affiliated with known bacterial phyla.

In all R-samples from the three CWs, the most abundant phylum was Proteobacteria (average abundance: 63.69–72.52% of total effective sequences), followed by Firmicutes (4.14–11.35%), Cyanobacteria (7.46–11.62%), and Bacteroidetes (3.15–12.15%). Regarding the S-samples, the most abundant phylum was Cyanobacteria (35.65% and 40.98%), followed by Proteobacteria (36.39% and 37.63%), Firmicutes (9.06% and 7.65%), and Verrucomicrobia (7.76% and 5.28%) in CWs treating wastewater with low and middle TAN concentrations; however, those were Proteobacteria (36.44%), Firmicutes (10.49%), and Bacteroidetes (9.39%) dominant in CWs treating wastewater with high TAN concentration. Furthermore, some dominant phyla exhibited statistical differences (Table S1, Supplementary Material). With regard to the R- and S-samples, statistical differences were noted among Proteobacteria, Cyanobacteria, Actinobacteria, and Verrucomicrobia (Student's *t*-test, *p* < 0.05). Regarding CWs with influents under different TAN concentrations, statistical differences were observed among Bacteroidetes, Thaumarchaeota, Verrucomicrobia, WS3, and Chlorobi (one-way ANOVA, *p* > 0.05). These results supported the abovementioned findings on spatial variation in microbial communities and the influence of TAN concentrations.

**Figure 4:**
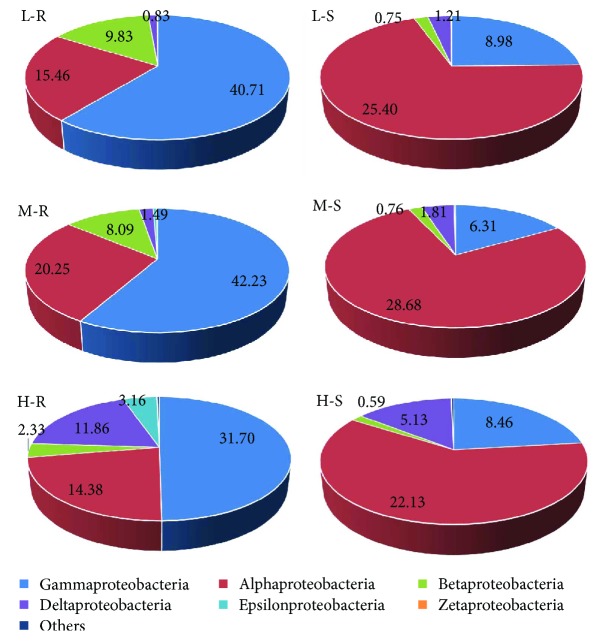
Relative abundance of Proteobacteria at the class level. Other Proteobacteria with relative abundance of <0.01% in each sample are included as “Others”.

Since Proteobacteria is a functionally and phylogenetically diverse phylum, it was further analyzed by class (Figure 4). In total, six well-recognized classes (Gammaproteobacteria, Alphaproteobacteria, Betaproteobacteria, Deltaproteobacteria, Epsilonproteobacteria, and Zetaproteobacteria) were observed, among which Gammaproteobacteria (31.70–42.23%) in the R-samples and Alphaproteobacteria (22.13–25.40%) in the S-samples were the top two most abundant classes.

Most of the phyla identified in this study have been discussed in the literature on their contribution to pollutant degradation [10, 20]. For example, Firmicutes, Bacteroidetes, and Actinobacteria have been reported to be the ubiquitous phyla in CWs and wastewater treatment processes, which are critical for the decomposition of contaminants [37, 38]. Verrucomicrobia are almost pervasive in soil [39], which explains its higher enrichment in the substrate than in the roots (Table S1, Supplementary Material). As a common wastewater treatment filamentous bacterium, the high relative abundance of Chloroflexi indicates its potential role in organic decomposition [40]. Moreover, previous studies have reported that many Planctomycetes can perform “anammox” metabolism [41]. Wang et al. [42] have concluded that high enrichment of Cyanobacteria is beneficial for maintaining high removal efficiency during summertime. In the present study, Cyanobacteria accounted for the largest proportion of the microbial communities in the S-samples, and its photosynthetic activity could produce oxygen (a key electron acceptor for pollutant-degrading bacteria) and organic exudates (key carbon source for heterotrophic bacteria) [43].

Proteobacteria are regarded as dominant in CWs treating wastewater [10, 20, 44] and in various rhizosophere systems [45, 46]. Microorganisms belonging to the phylum Proteobacteria are involved in the biodegradation of numerous pollutants, such as organic matter, nitrogen, and phosphorus [33, 47]. In the present study, Proteobacteria was the most abundant phylum in the R-samples and second largest phylum in the S-samples. At the class level, this study showed that Gammaproteobacteria dominated Proteobacteria in the R-samples, and Alphaproteobacteria was the most abundant class of Proteobacteria in the S-samples. For comparison, Urakawa et al. [33] reported that Alphaproteobacteria in plant rhizospheres and Gammaproteobacteria in substrate biofilms were the most abundant classes of Proteobacteria in a floating treatment wetland. Those inconsistent results between this study and the literature indicated that microbial communities could be affected by a number of factors, such as plant diversity [11], operation time [13], and wastewater quality characteristics [9].

The significant roles of archaea in water treatment have attracted intense attention in the literature, especially on their roles in nitrogen transformation [48]. In the present study, it was interesting to note that Thaumarchaeota, as the only detected archaea phylum, tended to be significantly richer in the CWs treating wastewater with low TAN concentrations (*p* < 0.05) (Table S1). This archaea group includes currently known ammonia-oxidizing archaea (AOA), such as *Nitrosopumilu*s and *Nitrososphaera*, which play an important role in nitrogen removal, especially the ammonia oxidation process [49]. When ammonia is a limiting resource for microbial growth, AOA were reported generally higher numbers in low ammonia environments as they are not limited by ammonia [50] concentrations in the low range.

### 3.4. Functional Genera and Their Relationships with Nutrient Removal

**Figure 5:**
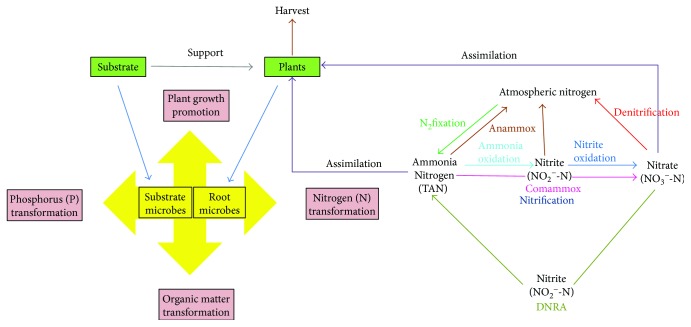
Key processes of recirculating CWs involved in the removal of various pollutants, especially marine nitrogen.

Analysis at the genus level allowed further verification of microbial diversity and relative abundance of genera in the R- and S-samples from CWs treating mariculture wastewater with different TAN concentrations (Figure S1). Microbial genus with a detection frequency of >1% in one or more samples was defined as a dominant genus. A total of 67 dominant genera (66 bacterial genera and 1 archaeal genus) were identified among the 18 samples, of which specific functional genera have been reported in the literature to play important roles in the key processes of CWs for the removal of various pollutants, especially marine nitrogen (Figure 5). The relative abundances of the functional genera in the R- and S-samples from CW receiving inflows with different concentrations of TAN were presented in Table 3.

**Figure 6:**
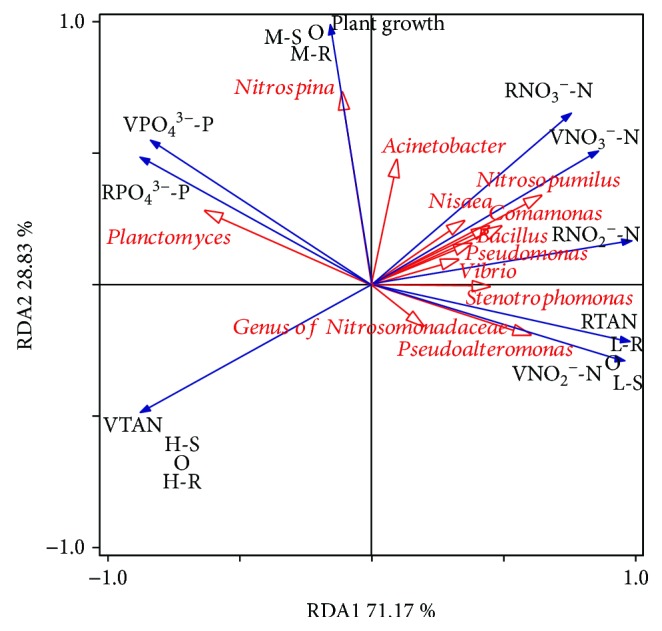
Redundancy analysis (RDA) biplot showing the relationship among functional genera in the sequencing data, nutrients' variation and removal rates, and plant growth. The first axis is horizontal, and the second axis is vertical. RTAN, RNO_2_^−^-N, RNO_3_^−^-N, and RPO_4_^3−^-P represent the removal rates of TAN, NO_2_^−^-N, NO_3_^−^-N, and PO_4_^3−^-P, respectively. VTAN, VNO_2_^−^-N, VNO_3_^−^-N, and VPO_4_^3−^-P denote the variation in TAN, NO_2_^−^-N, NO_3_^−^-N, and PO_4_^3−^-P after CW treatment, respectively.

In order to determine efficient microbial indicator, the relationships between the functional genera, nutrients' (TAN, NO_2_^−^-N, NO_3_^−^-N, and PO_4_^3−^-P) variation and removal rates, and even plant growth were evaluated by RDA biplot (Figure 6). As shown in Figure 6, the first and second axes explained 71.17% and 28.83% variation in the removal rates, respectively, which was consistent with all the other nutrients' variation, except for TAN. *Acinetobacter*, *Nisaea*, *Nitrosopumilus*, *Comamonas*, *Bacillus*, *Pseudomonas*, *Vibrio*, *Stenotrophomonas*, *Pseudoalteromonas*, and genus of Nitrosomonadaceae were positively correlated with the removal of nitrogen (Figure 6). Among them, *Vibrio* contributed most to the removal rate of NO_2_^−^-N, and *Pseudoalteromonas* were most related to the variation in NO_2_^−^-N. With regard to plant growth, *Nitrospina* had maximum effect, followed by *Acinetobacter*, *Pseudomonas*, and *Vibrio*. Besides, *Planctomyces* had a significant impact on phosphorus removal and variation in TAN.

While microbial communities have been proven to be influenced by different nutrient concentrations (such as TAN [36]), the established microbial communities, especially some functional microorganisms, can in turn affect nutrients' removal (Figure 6). Some microbial genera have been reported to directly participate in the nitrogen removal by ammonia oxidation, nitrification, and denitrification. For instance, *Acinetobacter* could transform nitrogen by heterotrophic nitrification and aerobic denitrification [51, 52]. And *Nisaea*, comprising two species, namely, the type species *Nisaea denitrificans* and *Nisaea nitritireducens* [53], can participate in denitrification and NO_2_^−^-N oxidation in nitrification, reducing NO_3_^−^-N and NO_2_^−^-N. Furthermore, *Nitrosopumilus* spp. and genus of Nitrosomonadaceae can oxidize ammonia [54–56] and contributed to ammonia removal in CWs. Besides, *Stenotrophomonas*, *Comamonas*, *Bacillus*, *Vibrio*, and *Pseudomonas* have also been reported to participate in the transformation of nitrogen [57–60]. *Pseudoalteromonas* has been shown to influence biofilm formation in various marine econiches [61–63] and could indirectly affect the removal of various pollutants such as NO_2_^−^-N. Similarly, in the present study, *Pseudoalteromonas* was noted to contribute most to the variation in NO_2_^−^-N. In fact, nitrite reduction is a challenging topic that researchers are dedicated to finding new solutions such as catalytic treatment [64]. The application of nitrite reduction bacteria *Pseudoalteromonas* could be a promising alternative.

Although rhizosphere is known to solubilize phosphorus through the chemical activity of root exudates and biological activity of rhizosphere bacteria, the underlying mechanisms are not yet clear enough. This study showed that *Planctomyces* had a significant impact on phosphorus removal. Similarly, Wu et al. [65] demonstrated that *Planctomyces* are positively correlated with available phosphorus content. Furthermore, *Planctomyces* has been reported to anaerobically oxidize ammonium (anammox) [66], which supports the finding of the present study that *Planctomyces* had a positive correction with the variation in TAN. *Nitrospina* are NO_2_^−^-N-oxidizing bacteria, which could transform NO_2_^−^-N to NO_3_^−^-N that can be easily taken up by plants [67, 68]. Interestingly, *Nitrospina* was noted to have maximum effect on plant growth in the present study. In consistent with the RDA results (Figure 6) in this study, Jha et al. [69] reported that *Pseudomonas* and *Vibrio* are *Salicornia* plant growth-promoting rhizobacteria, which can directly and indirectly improve the extent or quality of plant growth.

## 4. Conclusions

This study characterized the profile of microbial communities of three pilot-scale CWs treating mariculture wastewater under different TAN concentrations. The Illumina high-throughput sequencing results revealed a remarkable spatial variation in the diversity and composition of microbial communities between root and substrate in the CWs, which differed with the varying TAN concentrations in the mariculture wastewater. In particular, functional genera, such as *Nitrosopumilus* (archaea), *Vibrio*, *Pseudoalteromonas*, *Nitrospina*, and *Planctomyces*, were found to contribute to plant growth and effective removal of nitrogen and phosphorus from wastewater. The findings of this study could broaden the knowledge of the removal mechanism of contaminants in CWs and serve as a basis for the potential use of microorganisms as a biological indicator in CW management.

## Figures and Tables

**Table 1:** Characteristics of the influent and effluent of CWs treating mariculture wastewater under different TAN concentrations.

Parameters	TAN (mg/L)	NO_2_^−^-N (mg/L)	NO_3_^−^-N (mg/L)	PO_4_^3−^-P (mg/L)	pH	T (°C)
Final effluent						
L	0.020 ± 0.001^a^	0.008 ± 0.002^a^	1.348 ± 0.331^a^	0.420 ± 0.005^a^	7.88	19.9
M	0.773 ± 0.178^b^	0.006 ± 0.003^a^	1.145 ± 0.074^a^	0.398 ± 0.008^a^	7.72	19.9
H	3.510 ± 0.479^c^	0.013 ± 0.002^b^	0.675 ± 0.035^b^	0.356 ± 0.026^b^	7.62	19.8
Final variation						
L	−0.028 ± 0.001^a^	−0.187 ± 0.002^a^	−1.462 ± 0.331^a^	−0.008 ± 0.005	+0.04	+0.3
M	−0.323 ± 0.178^b^	−0.008 ± 0.003^b^	−1.210 ± 0.074^a^	−0.033 ± 0.008	+0.03	+0.5
H	−1.247 ± 0.479^c^	−0.005 ± 0.002^b^	−0.285 ± 0.035^b^	−0.026 ± 0.026	+0.10	+0.4
Removal rate (%)						
L	58.51 ± 2.13^a^	96.07 ± 1.04^a^	52.03 ± 11.77^a^	1.87 ± 0.012		
M	29.46 ± 16.21^b^	59.26 ± 19.25^b^	51.38 ± 3.13^a^	8.20 ± 0.019		
H	26.21 ± 10.07^b^	27.93 ± 10.92^c^	29.69 ± 3.65^b^	6.88 ± 0.068		

Final variation = effluent concentration − influent concentration; removal rate = ((effluent concentration − influent concentration)/influent concentration) × 100%. L: low influent TAN concentration group (0.75 mg/L); M: middle influent TAN concentration group (2.31 mg/L); H: high influent TAN concentration group (7.23 mg/L). Differences in the final effluent and removal rate among the groups were tested using one-way ANOVA. Different characters indicate significant differences (*p* < 0.05) (means ± SD, *n* = 3).

**Table 2:** Diversity estimation of the 16S rRNA gene libraries for the R- and S-samples.

Sample	OTU number		Chao 1 index		Shannon index		Simpson index	
	R	S	R	S	R	S	R	S
L	2007 ± 275	1492 ± 274	8704 ± 582	6947 ± 1464	7.41 ± 0.78	5.51 ± 1.20	0.96 ± 0.02	0.86 ± 0.08
M	1986 ± 268	1618 ± 246	9759 ± 1750^∗^	6557 ± 685^∗∗^	7.34 ± 0.85	5.70 ± 0.90	0.96 ± 0.03	0.86 ± 0.07
H	2089 ± 202	1698 ± 251	8338 ± 407	7234 ± 859	7.85 ± 0.71	6.50 ± 1.03	0.97 ± 0.02	0.91 ± 0.04

R: root samples; S: substrate samples; L: low influent TAN concentration group (0.75 mg/L); M: middle influent TAN concentration group (2.31 mg/L); H: high influent TAN concentration group (7.23 mg/L). Differences among the L, M, and H groups were tested using one-way ANOVA. Different characters indicate significant differences (*p* < 0.05). Differences between the R- and S-samples of each group were determined using Student's *t*-test. “∗” and “∗∗” indicate significant differences (*p* < 0.05) (means ± SD, *n* = 3).

**Table 3:** Relative abundances of some functional genera in the R- and S-samples from CWs treating mariculture wastewater with different TAN concentrations.

Microorganism	Function	Sample	L		M		H	
			Mean	SD	Mean	SD	Mean	SD
*Pseudoalteromonas*	Biofilm formation	R	0.0258	0.0390	0.0022	0.0007	0.0033^∗^	0.0019
		S	0.0007^ab^	0.0006	0.0020^a^	0.0010	0.0002^∗∗^^b^	0.0002
*Acinetobacter*	Denitrification, nitrification	R	0.0765	0.0519	0.1593	0.1105	0.0204	0.0225
		S	0.0003	0.0002	0.0001	0.0002	0.0001	0.0001
*Bacillus*	Plant growth promotion, denitrification, nitrification	R	0.0199	0.0074	0.0202	0.0116	0.0070	0.005
		S	0.0167	0.0131	0.0133	0.0028	0.0186	0.0261
*Pseudomonas*	Denitrification, plant growth promotion	R	0.0115^∗^^a^	0.0055	0.0090^∗^^ab^	0.0031	0.0034^b^	0.0019
		S	0.0019^∗∗^	0.0017	0.0018^∗∗^	0.0009	0.0024	0.0032
*Vibrio*	Plant growth promotion, nitrification	R	0.0167	0.0091	0.0216	0.0148	0.0080	0.0053
		S	0.0115	0.0184	0.0019	0.0013	0.0096	0.0007
*Stenotrophomonas*	Denitrification	R	0.0150^∗^	0.0104	0.0069^∗^	0.0050	0.0034^∗^	0.0050
		S	0^∗∗^	0	0^∗∗^	0	0^∗∗^	0
*Comamonas*	Denitrification	R	0.0123^∗^^a^	0.0034	0.0075^ab^	0.0048	0.0022^b^	0.0022
		S	0.0017^∗∗^	0.001	0.0032	0.0020	0.0016	0.0011
*Nisaea*	Denitrification, nitrite oxidation	R	0.0019	0.0011	0.0016	0.0013	0.0007^∗^	0.0002
		S	0.0002	0.0002	0.0003	0.0003	0^∗∗^	0
*Nitrospina*	Nitrite oxidation	R	0.0005	0.0002	0.0012	0.0005	0.0011	0.0008
		S	0.0009	0.0004	0.0016	0.0015	0.0002	0.0001
Genus of Nitrosomonadaceae	Ammonia oxidation	R	0.0001^a^	0.0001	0.0011^b^	0.0004	0.0016^b^	0.0006
		S	0.0051	0.0069	0.0022	0.0013	0.0024	0.0016
*Nitrosopumilus*	Ammonia oxidation	R	0.0145^a^	0.0020	0.0135^a^	0.0220	0.0003^b^	0.0003
		S	0.0063	0.0069	0.0020	0.0029	0	0
*Planctomyces*	Anammox, P solubilization	R	0.0045	0.0026	0.0058	0.0025	0.0071	0.0042
		S	0.0060	0.0042	0.0081	0.0018	0.0064	0.0053

SD: standard deviation; R: root samples; S: substrate samples; L: low influent TAN concentration group (0.75 mg/L); M: middle influent TAN concentration group (2.31 mg/L); H: high influent TAN concentration group (7.23 mg/L). Differences among the L, M, and H groups were tested using one-way ANOVA. Different characters indicate significant differences (*p* < 0.05). Differences between the R- and S-samples of each group were determined using Student's *t*-test. “∗” and “∗∗” indicate significant differences (*p* < 0.05).

## Supplementary Materials

Supplementary Materials Table S1: relative abundances of the major phyla in the R- and S-samples from recirculating CWs treating mariculture wastewater with different TAN concentrations. The values given are percentages of relative abundance. Figure S1: heatmap of microbial genera in the R- and S-samples from CWs treating mariculture wastewater with different TAN concentrations (dominant genus of relative abundance > 1%).
