# Supplementary material for: Characterization of Microbial Communities in Pilot-Scale Constructed Wetlands with Salicornia for Treatment of Marine Aquaculture Effluents
Source: Archaea. 2018 Apr 29;2018:7819840. doi: 10.1155/2018/7819840 (PMC5949191; doi:10.1155/2018/7819840)
Supplement: Supplementary Materials — Table S1: relative abundances of the major phyla in the R- and S-samples from recirculating CWs treating mariculture wastewater with different TAN concentrations. The values given are percentages of relative abundance. Figure S1: heatmap of microbial genera in the R- and S-samples from CWs treating mariculture wastewater with different TAN concentrations (dominant genus of relative abundance > 1%). [file 7819840.f1.pdf]

TABLE S1: Relative abundances of the major phylum in the root and substrate samples of the CWs treating mariculture wastewater of different TAN concentrations.

		L		M		H	
		Mean	SD	Mean	SD	Mean	SD
Proteobacteria	R	0.6690*	0.0832	0.7252*	0.0657	0.6369*	0.1080
	S	0.3639**	0.1250	0.3763**	0.0204	0.3644**	0.1138
Firmicutes	R	0.1135	0.0481	0.1076	0.0642	0.0414	0.0244
	S	0.0906	0.0666	0.0765	0.0168	0.1049	0.1360
Cyanobacteria	R	0.0948*	0.0199	0.0746*	0.0341	0.1162*	0.0249
	S	0.3898**	0.0947	0.4098**	0.0612	0.3565**	0.0378
Bacteroidetes	R	0.0545^{ab}	0.0183	0.0315^{a}	0.0119	0.1215^{b}	0.0575
	S	0.0248^{a}	0.0108	0.0282^{a}	0.0067	0.0939^{b}	0.0344
Planctomycetes	R	0.0212	0.0127	0.0175	0.0066	0.0194	0.0108
	S	0.0213	0.0076	0.0221	0.0089	0.0206	0.0132
Crenarchaeota	R	0.0146^{a}	0.0020	0.0137^{a}	0.0222	0.0003^{b}	0.0003
	S	0.0064	0.0069	0.0021	0.0030	0.0001	0.0001
Acidobacteria	R	0.0072	0.0075	0.0056	0.0029	0.0033	0.0024
	S	0.0066	0.0033	0.0078	0.0095	0.0022	0.0016
Actinobacteria	R	0.0056	0.0040	0.0028*	0.0006	0.0019	0.0010
	S	0.0069	0.0038	0.0055**	0.0008	0.0036	0.0012
Verrucomicrobia	R	0.0045^{*a}	0.0014	0.0030^{*a}	0.0004	0.0088^{b}	0.0020
	S	0.0776**	0.0401	0.0528**	0.0168	0.0249	0.0184
Chloroflexi	R	0.0044	0.0030	0.0079	0.0025	0.0120	0.0130
	S	0.0023	0.0026	0.0047	0.0009	0.0051	0.0041
WS3	R	0.0004^{a}	0.0002	0.0003^{a}	0.0002	0.0084^{b}	0.0059
	S	0.0005	0.0003	0.0002	0.0001	0.0071	0.0072
Chlorobi	R	0.0002^{a}	0.0001	0.0014^{a}	0.0008	0.0111^{b}	0.0075
	S	0.0001^{a}	0.0001	0.0005^{a}	0.0002	0.0039^{b}	0.0017
others	R	0.0010	0.0055	0.0088	0.0026	0.0187	0.0140
	S	0.0093	0.0057	0.0134	0.0086	0.0125	0.0085

R: Root samples; S: Substrate samples. Difference among groups of L, M, H groups were tested with one-way ANOVA and different characters indicate significant differences ($p < 0.05$). Difference between R and S samples in each group were tested with Student's t test and '*', '**' indicate significant differences ($p < 0.05$). (means ± SD, n = 3)

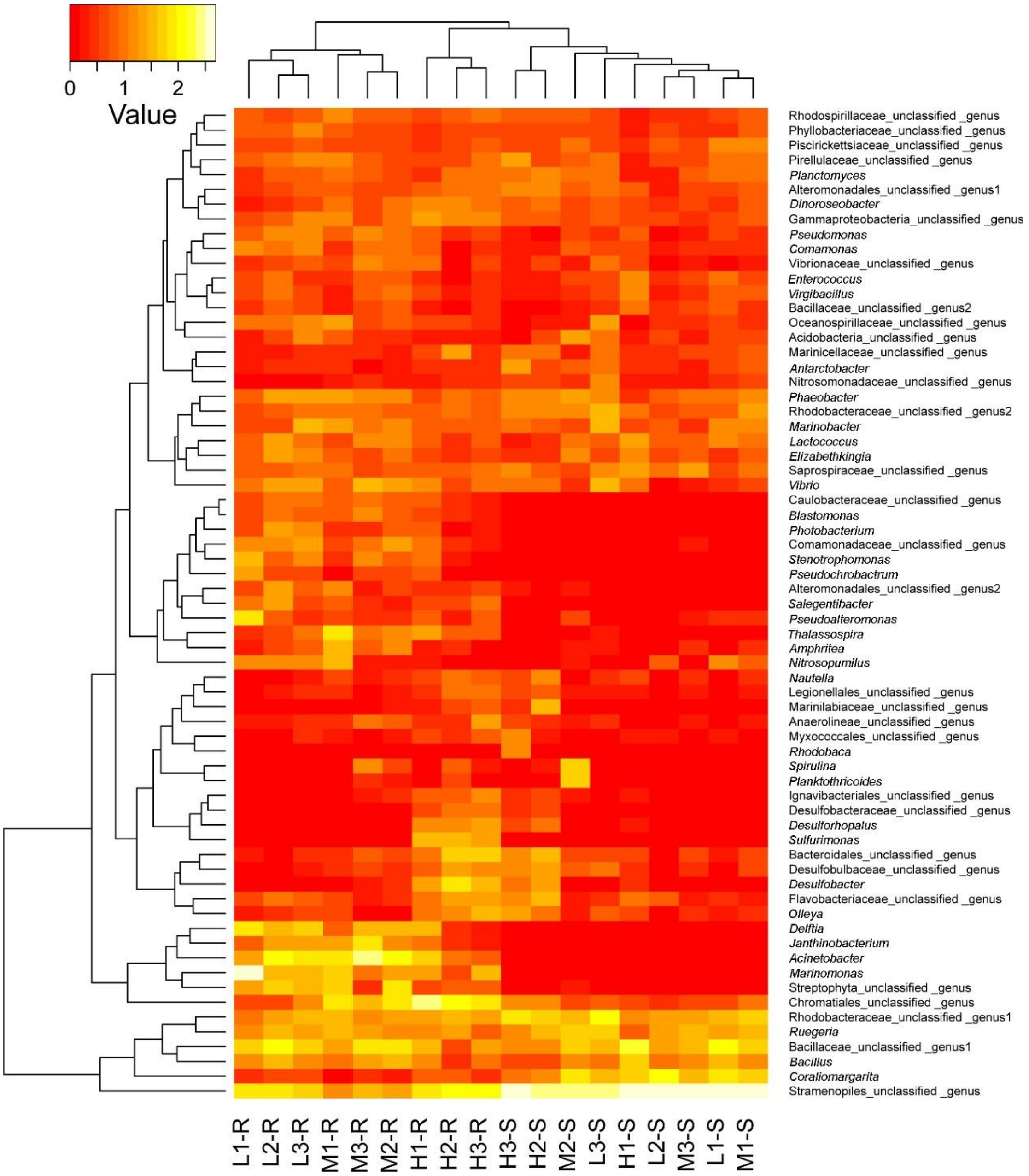

FIGURE S1: Heatmap of microbial genera of root and substrate samples treating mariculture wastewater of different TAN concentration. (dominant genus of relative abundance >1%).
